# Adaptation strategies in haloalkaliphilic fungi: *Aspergillus salinarum*,* cladosporium sphaerospermum*,* and penicillium camemberti*

**DOI:** 10.1186/s12866-025-03848-1

**Published:** 2025-03-21

**Authors:** Noura I. Farouk, Shadia M. Sabry, Asmaa M. Elhosainy, Magda A. El-Meleigy

**Affiliations:** Botany and Microbiology Department, Faculty of Science (Girls), Al-Azhar University, Nasr City, 11754 Cairo Egypt

**Keywords:** Haloalkaliphilic fungi, *Aspergillus salinarum*, *Cladosporium sphaerospermum*, *Penicillium camemberti*, Stress adaptation, Organic acids, Antimicrobial activity

## Abstract

**Background:**

Extremophilic fungi thrive in extreme environments, revealing life’s origins and enhancing biodiversity while offering insights into evolutionary biology. This study investigates the adaptation mechanisms of haloalkaliphilic fungi *Aspergillus salinarum*, *Cladosporium sphaerospermum*, and *Penicillium camemberti*, isolated from Egyptian soils, adapted to life under extreme conditions of high salt (15%) and alkaline pH (10). These properties make them interesting for fundamental research and the exploration of biotechnological potential.

**Results:**

These fungi exhibited increased levels of soluble proteins and lipids in cell-free extracts under stress conditions. Enzyme activities, specifically peroxidase and tyrosinase, were significantly induced, with maximum induction varying by species and incubation time. Significant amounts of organic acids, including citric, oxalic, and butyric acids, were detected in higher quantities under extreme conditions, with total organic acid content increasing by up to 2.97%. The culture filtrates demonstrated enhanced antimicrobial activity against various Gram-positive and Gram-negative bacteria, *Bacillus Subtilis* (ATCC 6633); *Staphylococcus aureus* (ATCC 6538); *Escherichia coli* (ATCC 8739); *Pseudomonas aeruginosa* (ATCC 90274); yeast, *Candida albicans*, but not against *Aspergillus niger*.

**Conclusions:**

These findings highlight the potential industrial applications of these fungi in biotechnology and pharmaceuticals due to their biochemical responses and antimicrobial properties.

## Introduction

Extremophilic microorganisms, capable of surviving in extreme natural or artificial habitats, are categorized into thermophilic, psychrophilic, acidophilic, alkaliphilic, halophilic, and barophilic types [1]. These microorganisms, found in environments with physicochemical parameters at the limits of life, belong to bacteria, archaea, fungi, and algae. Their adaptation to extreme conditions offers insights into life’s evolution and potential breakthroughs in life sciences [2, 3].

Numerous reviews have extensively examined the mechanisms that enable haloalkaliphilic fungi to adapt to extreme environments, as well as the specificity of their enzymes for a wide range of commercial and environmental applications [4, 5]. These fungi are equipped with a diverse array of biocatalysts that allow them to thrive under high salinity and alkaline pH conditions [6, 7]. Among the most studied enzymes are amylase, peroxidases, and proteases [8–11]. Also, the haloalkaliphilic bacterium, *Halobiforma* sp. strain BNMITR, isolated from a soil sample of Sambhar Lake, Northern India, was found to produce an extracellular alkaline protease [12].

The distinct characteristics of these enzymes, such as their stability under extreme physicochemical conditions like high hydrogen ion and NaCl concentrations [8, 13, 14], underscore the urgent need for alternative compounds in various industries [4]. Marine microorganisms, owing to their vast genetic and biochemical diversity, represent promising sources for industrially applicable enzymes [15].

Haloalkaliphilic fungi serve as excellent biological agents for soil myco-remediation. These fungi, found in saline-alkaline soils, produce high levels of enzymes that contribute to salt and alkali resistance, including bilirubin oxidase from *Myrothecium* sp. IMERI [16], alkali-stable endoglucanases B from *A. niger* BCRC31494 [17], and alkaline xylanase from *A. nidulans* KK-99 [18]. These and other molds have a significant role in biodegradation of organic material in saline-alkali soils.

A previous study noted that the production of organic-mineral compounds is facilitated by organic acid release [19]. A further elaboration submitted that these organic acids, upon release, generate protons that acidify the alkaline soil solution [20]. Saline-alkaline soils typically lack readily available phosphorus, and these researchers proposed that organic acids might reduce soil solution pH through acid-base neutralization, thereby aiding in the solubility of several inorganic phosphorus compounds in the rhizosphere.

The enzymes known as halophilic proteases are responsible for the hydrolysis of peptide bonds found in proteins. They are widely used in the leather industry to dehair the skin. Also play a significant part in the bioremediation process, which treats wastewater that is extremely salinized and in the detergent industry because they improve the detergent’s ability to remove protein stains. *Nocardiopsis prasina* produces salt-stable proteases that are active in the pH range of 7–10 and the temperature range of 20–42 ^0^C [21].

In a study by [22], 52 strains were isolated from Lake Magadi in Kenya, predominantly from the genera *Aspergillus*, *Penicillium*, *Cladosporium*, *Phoma*, and *Acremonium*. Their cell-free extracts and crude extracts demonstrated inhibitory effects on a variety of fungal plant pathogens, such as *Schizophyllum commune*,* Aspergillus fumigatus* strain EG11-4, *Cladosporium halotolerans* CBS 119,416, *Phoma destructive*, and *Didymella glomerata*, as well as *Bacillus subtilis*, *Escherichia coli*,* Pseudomonas aeruginosa*,* Salmonella* spp., *Shigella* spp., *Candida albicans*, and other fungal plant pathogens.

Current work aimed at the identification of the strategy of haloalkaliphilic fungi *Aspergillus salinarum*,* Penicillium camemberti*, and *Cladosporium sphaerospermum* in adaptation at high salt and pH media which contributes to advancing research on extremophiles.

Our findings revealed that the three fungal isolates are the future sources of salt-tolerant transgenic for agriculture, and on the treatment of saline wastewater and saline soil treatment due to their adaptability for salt and high pH stress.

## Materials and methods

### Fungal isolates

Haloalkaliphilic fungal isolates *A. salinarum* (oblige halophilic fungus didn’t grow on medium devoid of added salt, it needs at least 5% potassium chloride (KCl) for growth), *P. camemberti* and *C. sphaerospermum* were previously isolated from Egyptian soils and identified morphologically and genetically as clarified in [11].

### Growth of haloalkaliphilic fungal isolates

Modified Dox, s medium [11] was used for this purpose, the pH of the medium was adjusted at 10 using (10.0% Na_2_CO_3_ autoclaved separately and added to the medium after autoclaving) and 15% KCl was added before autoclaving. In each case triplicate sets of 250 ml conical flasks were sterilized, then flasks inoculated with 2 discs of each fungal isolate (separately) and incubated at 28 ± 2ºC.

### Preparation of cell-free extract

Cultures were grown under two distinct conditions: one in a liquid medium containing 15% KCl with a pH of 10 (extreme conditions), and the other in a liquid medium without potassium chloride at a pH of 7 (neutral conditions). The flasks were inoculated and incubated at 28 ± 2 °C for 10 days to prepare cell-free extracts. Mycelia were then harvested, washed with buffer, and ground with an approximately equal volume of clean, washed sand in a cold mortar. The extraction was performed using sodium phosphate buffer at pH 7.1 for neutral conditions (pH 7 and no salt), and glycine-NaOH buffer for extreme conditions (pH 10 and 15% salt). The final slurry was centrifuged for ten minutes at 6000 rpm at room temperature, and the supernatant was taken out for biochemical examination.

#### Protein determination

Carried out using [23] by utilizing egg albumin standard protein, and a spectrophotometer was used to read the appeared color at 720 nm. Spekoll II.

#### Determination of consumed sugar

This was carried out using the anthrone reagent method as described by [24].

#### Lipids determination

Lipids determination was carried out using AOAC official method No 920.194 the data are expressed in µg/l (ppm) using an Atomic Absorption Spectrophotometer (model: GBC932AA) at R C M B.

#### Assay of peroxidase enzyme

Peroxidase activity was determined by the method of [25].

#### Assay of tyrosinase enzyme

Tyrosinase activity was measured using the procedure described by [26].

To define one unit of enzyme activity, the change in absorbance unit per minute per milliliter of the enzyme was computed.

### Determination of organic acids

Organic acids were determined in culture filtrate of haloalkaliphilic fungal isolates by using High-Performance Liquid Chromatography (HPLC) Euchromatic Mod.C 1620 Liquid Chromatography at Regional Center for Mycology and Biotechnology, Al-Azhar University, Cairo, Egypt (R.C.M.B) Software PA station 2015. Flow Rate: 0.5 ml/min DEECTOR: 210 nm. Mobile phase: 0.5% Orthophosphoric acid H_3_PO_4_ (dissolved in ultrapure deionized water). Injection vol: 10 micro/l. Two culture conditions were used at neutral (pH 7 and salt-free medium except *A. salinarum* at 5% KCl) and at extreme conditions (pH 10 and 15% salt).

### Screening the antimicrobial activity of haloalkaliphilic fungal isolates

Two conditions of cultivation were used, the first one containing 15% potassium chloride and pH 10 (extreme conditions), and the second was sodium chloride-free medium at pH 7 (neutral conditions). Flasks were inoculated and incubated at 28 ± 2 ^o^C for 3–5 days in an incubating rotary shaker at 150 rpm. And then left it for use. Filtrates were used in this experiment. The Agar well diffusion method was used. Nutrient agar medium (for bacteria) and Dox, s medium (for fungi) [27]. Test organisms used; Gram-positive bacteria: *Bacillus Subtilis* (ATCC 6633) and *Staphylococcus aureus (ATCC 6538)*; gram-negative bacteria: *Escherichia coli (ATCC 8739)* and *Pseudomonas aeruginosa (ATCC 90274)* and fungi: *Candida albicans* and *Aspergillus niger.*

### Statistical analysis

The statistical analysis was performed using one-way analysis of variance (ANOVA), followed by post-hoc tests if *P* < 0.05. The analysis was conducted using the GraphPad InStat 3.06 software.

## Results

### Total soluble proteins, carbohydrates, and lipids in cell-free extracts of polyextremophilic fungal isolates at different growth conditions

**Fig. 1:**
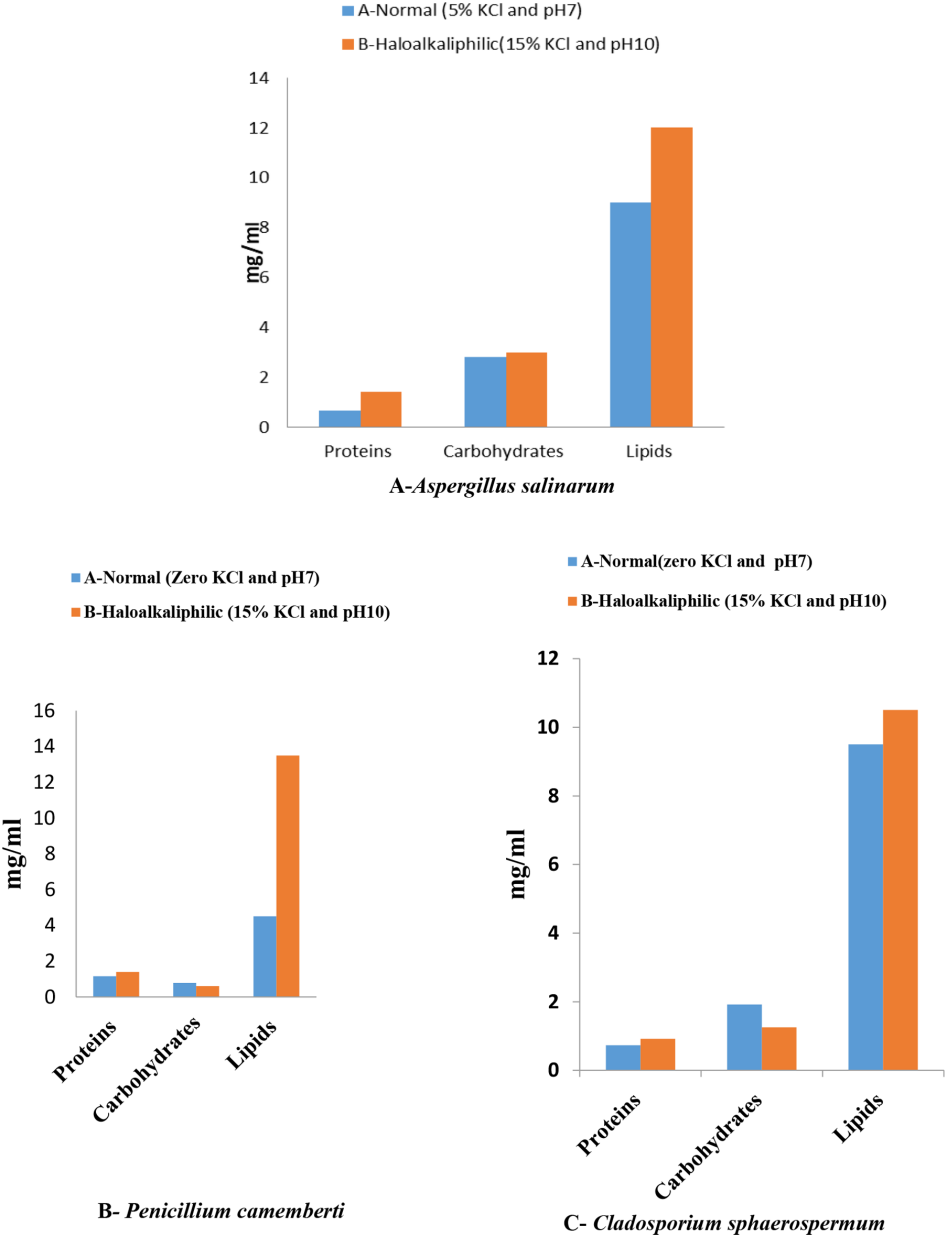
Total soluble proteins, carbohydrates, and lipids in cell-free extracts of polyextremophilic fungal isolates *A. salinarum*,* P. camemberti*, and *C. sphaerospermum* at different growth conditions

Haloalkaliphilic conditions increased the content of total soluble proteins in cell-free extracts to the three fungal isolates, *A. salinarum*,* P. camemberti*, and *C.sphaerospermum*, comparing that found in neutral conditions (Fig. 1A, B, and C). Contents of total soluble proteins, carbohydrates, and lipids in cell-free extracts of *A. salinarium* were higher in extreme conditions than the amounts found in the cell-free extracts in neutral conditions (Fig. 1A). The figure in the case of *Penicillium camemberti* and *Cladosporium sphaerospermum* was different to some extent, the amounts of total soluble proteins and lipids were increased in extreme conditions while that of total soluble carbohydrates were decreased in extreme conditions compared to that found in cell-free extracts in neutral conditions (Fig. 1B and C respectively).

### Activity of peroxidase and tyrosinase enzymes secreted by fungal isolates A. salinarum, P. camemberti, and C. sphaerospermum on neutral and haloalkaliphilic conditions

**Fig. 2:**
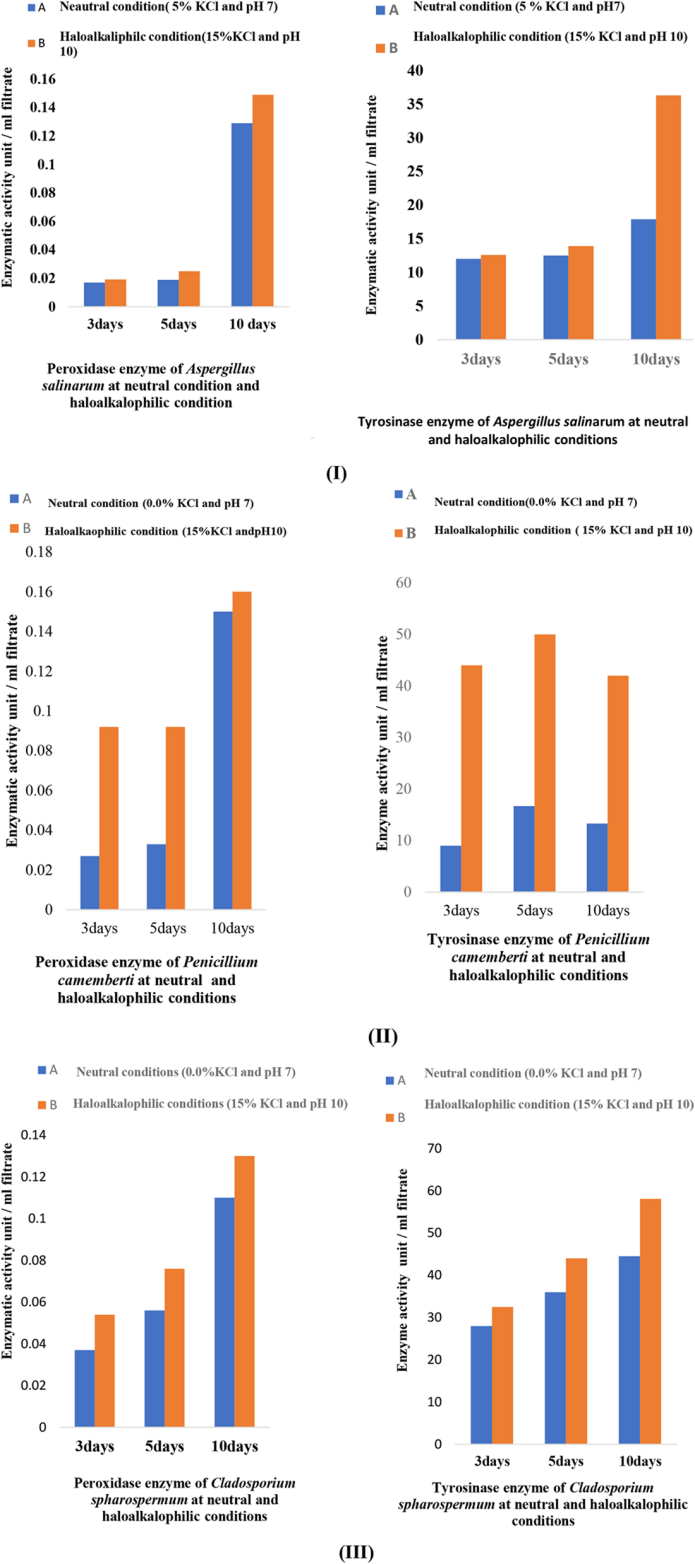
Effect of incubation conditions (neutral and haloalkaliphilic) on induction of peroxidase and tyrosinase enzymes by fungal isolates *A. salinarum* (I*)*,* P. camemberti* (II) and *C. sphaerospermum* (III)

The induction of peroxidase and tyrosinase enzymes Fig. 2 (I, II, and III), was increased by increasing incubation time from 3 to 5 days and 10 days with the exaptation of tyrosinase for *P. camemberti* its maximum induction after 5 days.

### Production of organic acids by haloalkaliphilic fungal isolates at different growth conditions

**Fig. 3:**
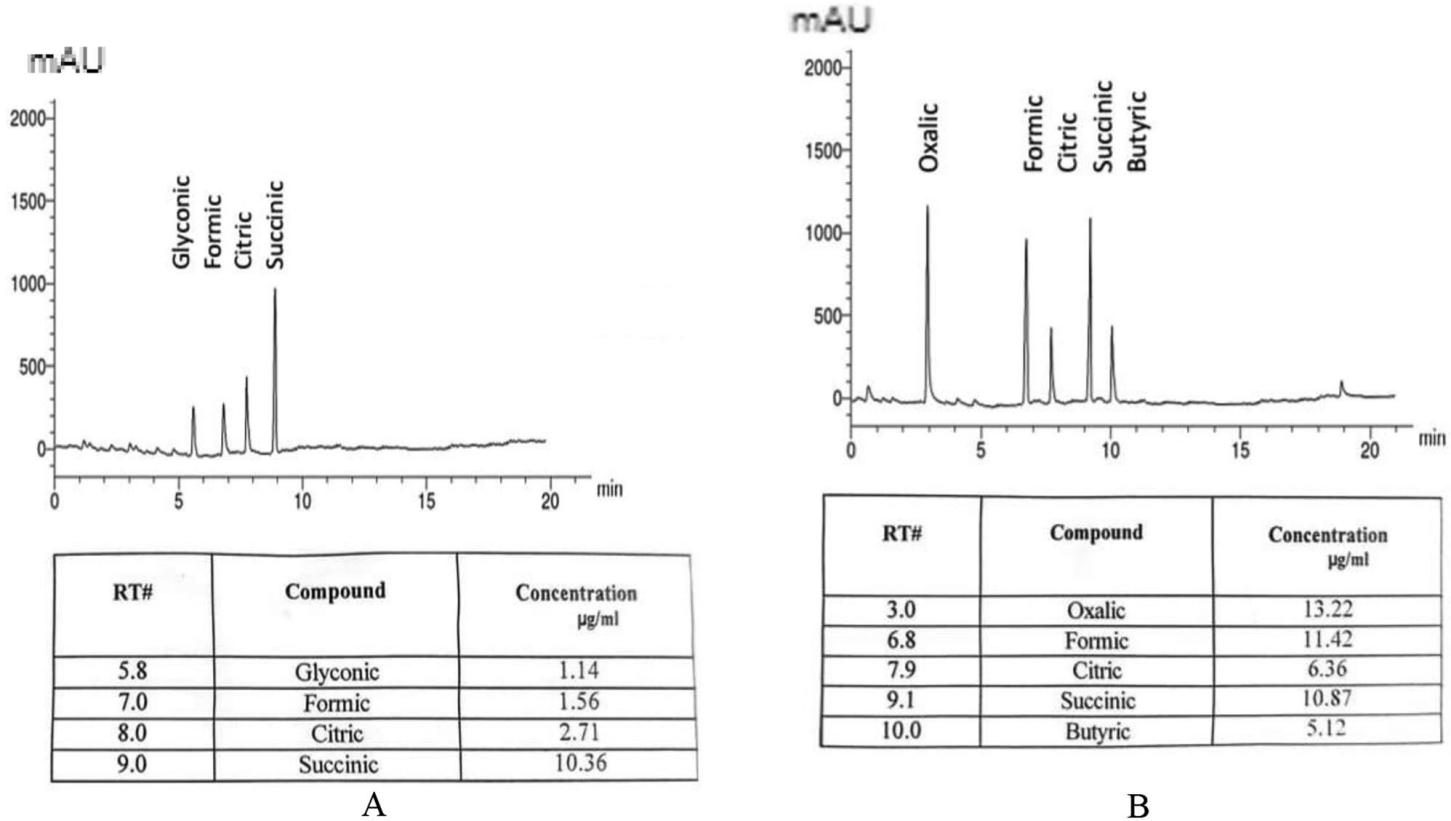
Organic acids production in culture filtrate by *Aspergillus salinarum* A, at neutral conditions (pH 7 & 5% KCl) and B at extreme conditions (pH 10 & 15% KCl)

**Fig. 4:**
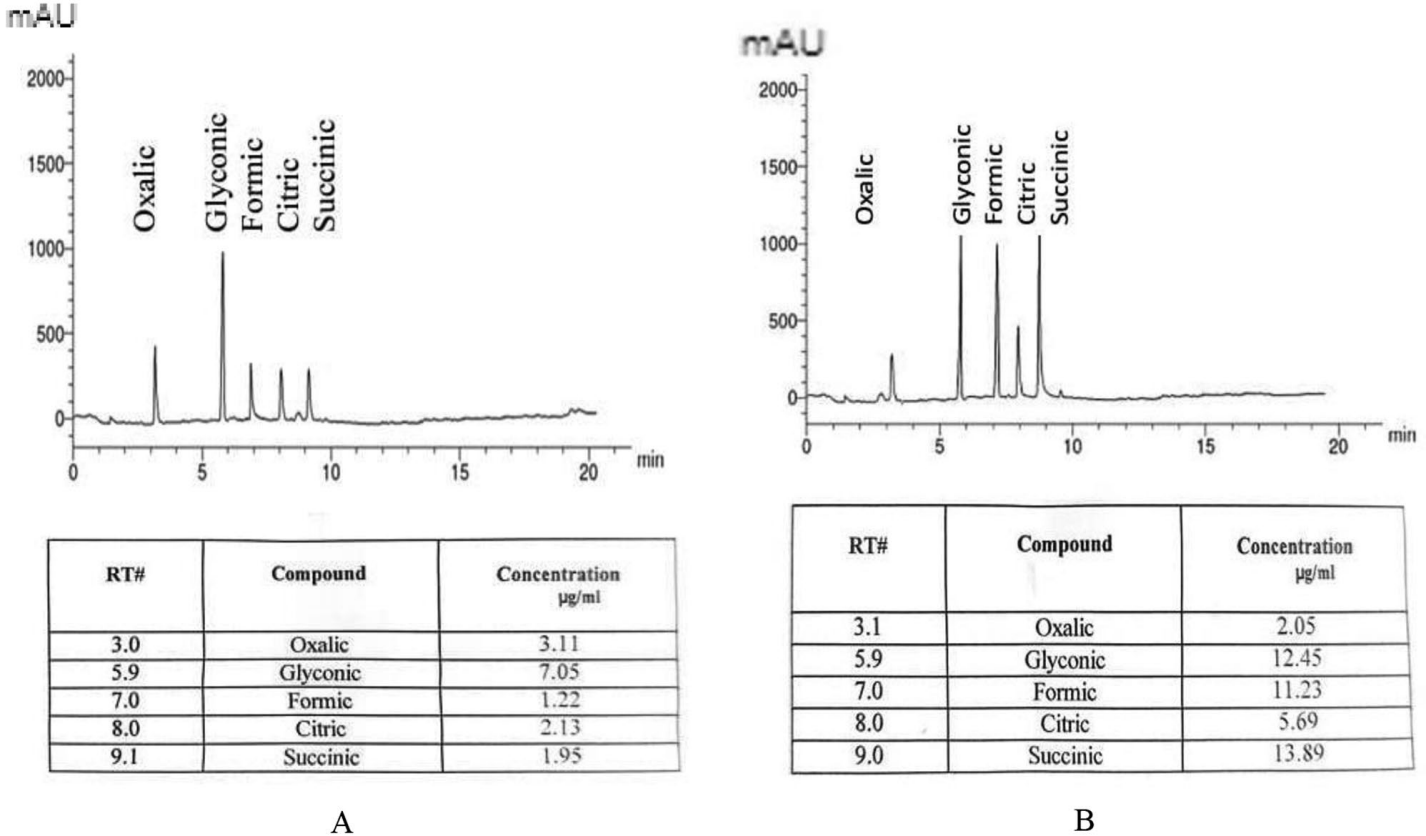
Organic acids production in culture filtrate of *Penicillium camemberti* A, at neutral conditions (pH7 & 0.0% KCl) and B, at extreme conditions (pH10 and 15% KCl)

Significant amounts of organic acids were revealed in culture filtrates of *A. salinarum*,* P. camemberti*, and *C. sphaerospermum* in haloalkaliphilic and neutral conditions. Figure 3 demonstrated that oxalic, formic, citric, succinic, and butyric acids were detected in culture filtrates of *Aspergillus salinarum* in extreme conditions only, while glyconic was detected only in neutral conditions. The amount of total organic acids was increased by 2.97% in extreme conditions. *P. camemberti* contains oxalic, citric, succinic, and butyric acids in a higher amount in extreme conditions than in neutral conditions (Fig. 4). Also, the amount of total organic acids was increased by 2.93% in extreme conditions.

**Fig. 5:**
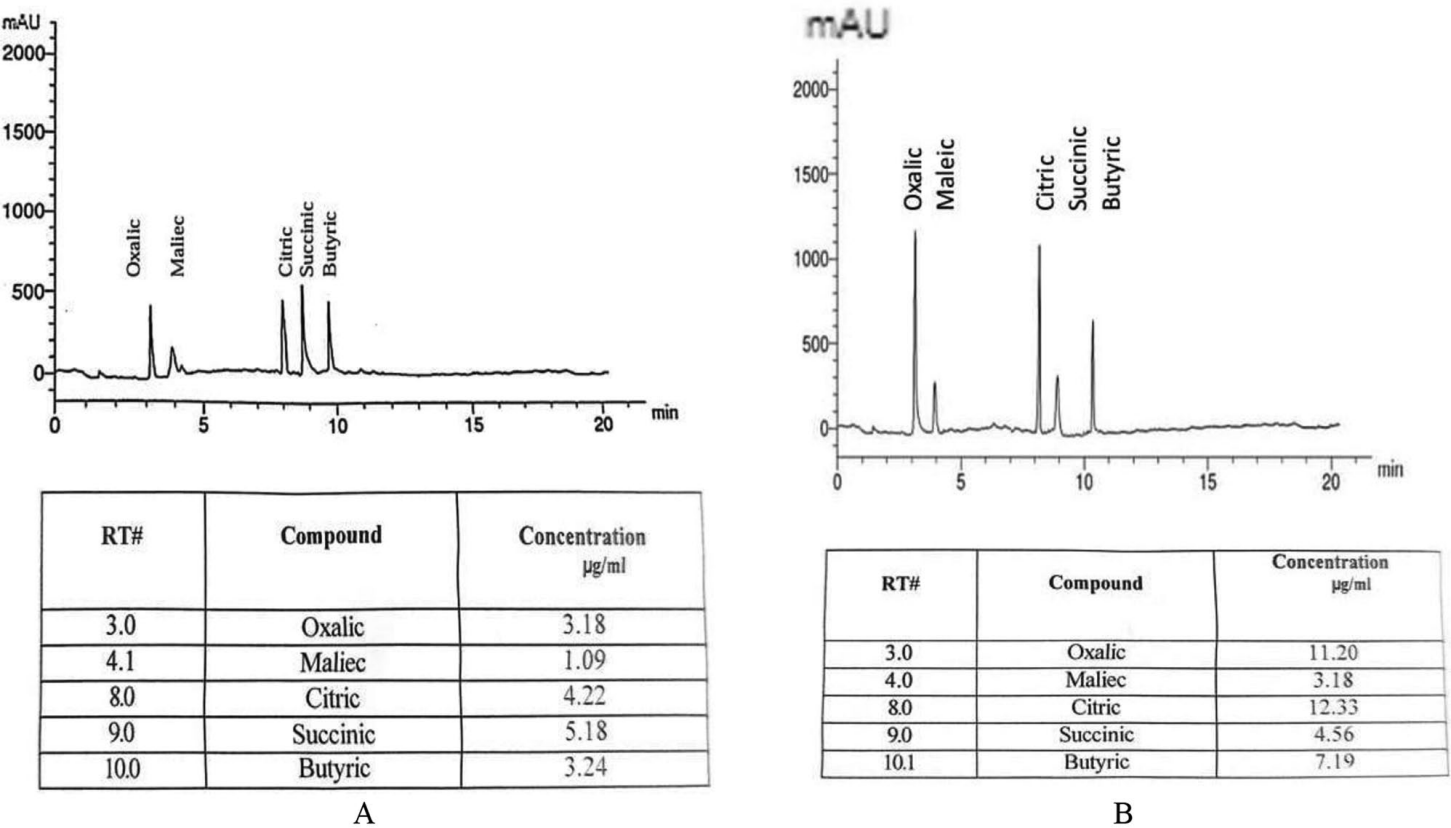
Organic acids production in culture filtrate of *Cladosporium sphaerospermum* A, at neutral conditions (pH7 and 0.0% KCl) and B, at extreme conditions (pH10 and 15% KCl)

In *C. sphaerospermum* maleic acid was detected in extreme and neutral conditions while glyconic and formic were not detected (Fig. 5). Higher quantities of oxalic, citric, and butyric were detected in filtrates on haloalkaliphilic conditions than that found in neutral conditions. The amount of succinic acid was decreased in extreme conditions than that of neutral ones. Generally, the total organic acids concentration in extreme conditions was higher than that found in neutral ones, they increased by 2.27%.

### Screening the antimicrobial activity of haloalkaliphilic fungal isolates

Table 1 illustrates the effect of antimicrobial activity of filtrates of *Aspergillus salinarum*,* Penicillium camemberti*, and *Cladosporium sphaerospermum* grown at neutral conditions (pH 7 and 0.0% (w/v) potassium chloride for the eventuality of *Penicillium* and *Cladosporium* and 5% (w/v) potassium chloride in case of *Aspergillus* while haloalkaliphilic conditions (pH 10 and 15% (w/v) potassium chloride after 3 days and 5 days of incubation, they screened against test organisms using agar well diffusion method. Gram-positive and Gram-negative bacteria, *Bacillus Subtilis* (ATCC 6633); *Staphylococcus. aureus* (ATCC 6538); - *Escherichia coli* (ATCC 8739); *Pseudomonas aeruginosa* (ATCC 90274); yeast, *Candida albicans*, and a fungus *Aspergillus niger* were utilized as test organisms in this study. The three fungal isolates filtrates had no effect against *Aspergillus niger* as a test organism.

Haloalkaliphilic conditions enhanced the antimicrobial activity of the three fungal isolates which appear from the results in Table 1. The induction of the antimicrobial agents was increased by increasing incubation time from 3 to 5 days, except that of *Aspergillus salinarum*, the induction after 3 days was lower than that at neutral conditions of incubation and it has no antimicrobial activity against *Pseudomonas aeruginosa* (ATCC 90274) at neutral condition after 3 days but after 5 days the activity appeared. The antimicrobial activity of the broth medium used in extreme conditions at (15% (w/v) KCl and pH 10 was tested against test organisms, negative result was obtained.

**Table 1:** Screening the biological activity of polyextremophilic fungal isolates *Aspergillus salinarum*,* penicillium camemberti*, and *Cladosporium sphaerospermum* cultivated on different conditions

Isolates	*A. salinarum					*P*. camemberti						C. sphaerospermum					
Incubation time (Days)	3		5		C	3		5		C		3		5		C	
Growth Conditions	*N*	E	*N*	E	C	*N*	E	*N*	E	C	*N*		E	*N*	E	C	
**Test organisms**	**Inhibition zones are represented as mm.**																
***Bacillus Subtilis*** **(ATCC 6633)**	10	23	21	30	23	21	24	20	26	24	18		25	23	27	25	
***Staphylo.aureus (ATCC 6538)***	11	20	19	25	20	18	21	18	23	20	16		20	18	25	15	
***Escherichia coli (ATCC 8739)***	10	16	15	20	15	14	16	14	17	14	13		15	14	16	15	
***Pseudomonas aeruginosa (ATCC 90274)***	NA*	19	16	24	18	15	15	15	20	19	15		17	19	22	16	
***Candida albicans (ATCC 10221)***	13	24	21	28	23	23	26	21	27	23	18		22	21	26	22	
***Aspergillus niger***	NA	NA	NA	NA	18	NA	NA	NA	NA	17	NA		NA	NA	NA	20	

NA*: No activity C = Control: Gentamycin and Fluconazole for fungi

N = Neutral condition (0.0% KCl and pH 7) E = Extreme condition (15% KCl and pH 10)

*Neutral condition (5% KCl and pH 7) for *A. salinarum*

## Discussion

The ability of fungi to sense and respond to external stimuli is critical for survival. Salt-tolerant fungi employ various mechanisms to adapt to high salinity environments, including alterations in the architecture of the cell wall, production of small amphipathic extracellular proteins [28], and the elimination of salts as well as the production or accumulation of compatible organic solutes. Changes in pH exert significant stress on cellular functions, impacting micronutrient availability, protein function, and membrane potential [29].

A study of [30] observed the accumulation of trehalose, mannitol, and arabitol in two obligate alkaliphilic fungi, *Sodiomyces magadii*, and *S. alkaline* [31, 32]. discussed various adaptation mechanisms to stress, highlighting the accumulation of cytoprotective compounds such as carbohydrate osmolytes. These include cytosolic carbohydrates like trehalose and mannitol, as well as phosphatidylcholines (PC), sterols, and triglycerides in the mycelium of the alkalophilic fungus *Sodiomyces alkalinus*. Additionally, they noted modifications in the composition of membrane lipids.

A previous investigation recorded an increase in total lipids in three halophilic fungi with rising NaCl concentrations [33]. Similarly, several studies documented increases in protein and carbohydrate content in halophilic fungi [34–36]. In another report, a significant rise in intracellular proteins, carbohydrates, and lipids when sodium chloride concentration was increased in the growth medium of *T. piluliferum* fs. Halophila AZ and *Aspergillus restrictus* [37].

Because extremophilic archaea develop in harsh environments, they are one of the perfect candidates to carry out biocatalytic tasks. Their production of proteins and enzymes that are active has transformed industrial biotechnology. The food, pharmaceutical, paper, leather, and textile industries are a few that have profited. However, relatively few enzymes from Archaea are commercially available; the majority are bacterial or fungal. However, their ability to survive in environments where bacterial and fungal enzymes are denatured is currently a focus. Proteases, lipases, amylases, cellulases, and other archaeal enzymes have already undergone testing [38].

It was recorded that fungi such as *Aspergillus*, *Fusarium*, *Penicillium*, *Alternaria*, and *Rhizopus* produce peroxidase and laccase enzymes [25, 39]. Also, extremophilic bacteria involving genus *Acidithiobacillus*,* Arthrobacter*,* Bacillus*,* Caldicellulosiruptor*,* Clostridium*,* Coprothermobacter*,* Enterobacter*,* Geobacillus*,* Micrococcus*,* Paenibacillus*,* Penicillium*,* Picrophilus*,* Pseudoalteromonas*, and *Thermobifida* secreted enzymes such as α-amylase, subtilase, β-galactosidase, xylanase, β-glucosidase, decarboxylase, endoglucanase, dehydrogenase, tetrathionate hydrolase [40].

The production of manganese peroxidase by the marine-derived strain *Mucor racemosus* CBMAI 847 is likely influenced by salt concentration [41]. Similarly, *Alternaria alternata* ANF238 was identified as a promising candidate for lignin peroxidase production through solid-state fermentation, highlighting its potential for various industrial and biotechnological applications [42]. The application of the peroxidase in the biosensing of hydrogen peroxide, glucose, pesticides, and herbicides as well as blood components such as cholesterol, urea, dopamine, and xanthine have been extensively reviewed by [43]. Also, peroxidase application of the biocatalysts in wastewater treatment through degradation of dyes, pesticides, and other organic compounds has been applied [43].

Fungi are renowned for their capability to degrade persistent pollutants, including those found in textile dyes [44]. Due to their adaptation to high salt and pH levels, marine fungi exhibit a significant biological advantage in effluent decolorization and degradation, particularly in saline and alkaline conditions typical of many textile processes. According to [45], fungal cells decolorize dyes through oxidative processes, resulting in non-toxic derivatives. The ligninolytic system, one of the extracellular enzymes produced by filamentous fungi, plays a crucial role in environmental cleanup [46]. A study reported the production of enzymes such as laccases, peroxidases, and manganese peroxidase by halophilic marine fungi, highlighting their ability to degrade lignocellulose [47].

Tyrosinase’s industrial applications have garnered a lot of interest. It is utilized as a key catalytic enzyme in the food and cosmetics sectors as well as in the pharmaceutical industry to produce L-DOPA. Applications for the melanin pigment produced by tyrosinase include immunogens, drug carriers, antioxidants, antiviral agents, and radiation protection [48].

Soil microorganisms are integral to the solubilization of various inorganic phosphorus compounds through the release of organic acids and other compounds in the rhizosphere [20]. Moreover, these organic acids also lower soil solution pH via acid-base neutralization. Previously, hydrogen ion concentration increased beyond pH 8.0, *Aspergillus glaucus* enhances its production of a range of organic acids, including malic, oxalic, and citric acids [49].

A multitude of secondary metabolites, including antimicrobial antibiotics, pigments, and toxins have been announced to be produced by fungi and actinomycetes [50–52]. Investigated fungal genera known for secondary metabolite production include *Fusarium*, *Aspergillus*, *Acremonium*, and *Penicillium*. Other findings revealed that active compounds from the secondary metabolites of *Nocardiopsis* sp. AJ1, isolated from solar salterns in India, effectively suppressed pathogenic bacteria, fungi, and viruses, offering the potential for antimicrobial development [53]. Furthermore [22], reported that *Penicillium chrysogenum* exhibited notable antimicrobial activity under haloalkaliphilic conditions against human enteric pathogens *Bacillus subtilis*, *Escherichia coli*, *Pseudomonas aeruginosa*, *Salmonella* spp., *Shigella* spp., and the fungal human pathogen *Candida albicans*. A recent investigation of [54] highlighted the potential of halophilic enzymes in microbiological testing, sterility testing within the antibiotic industry, and in evaluating the sensitivity against β-lactamases by novel antibiotics.

## Conclusions and remarkable

The three haloalkaliphilic fungal isolates *A. salinarum*,* P. camemberti*, and *C. sphaerospermum* had many strategies for adaptation they increased total soluble proteins, carbohydrates, and lipids in their cells, increased the production of tyrosinase and peroxidase enzymes, duplicate the organic acids three times in their filtrates also they produced antimicrobial agents affect most pathogenic bacteria. All these points require more work to be visualized. These organisms may be used in large-scale production of single-cell proteins, antimicrobial agents, and saline-alkali soil mycoremediation. Also, their genes may be used too.

## Data Availability

The datasets generated during and/or analyzed during the current study are available from the corresponding author and was described carefully. To be clear the results of the study didn’t involve human or animal samples.

## Declarations

### Abbreviations

All abbreviations are mentioned in full form the first time they appear in the manuscript.
